# Clinical Severity and Systemic Inflammatory Indices as Predictors of In-Hospital Mortality After Limb Amputation a Retrospective Cohort Study

**DOI:** 10.3390/jcm14228063

**Published:** 2025-11-14

**Authors:** Alim Namitokov, Tarlan Bakhishev, Roman Vinogradov, Aslan Zakeryaev, Sultan Butaev, Eldar Urakov, Gerey Khangereev, Leonid Sakhno, Marina Pchegatluk, Dmitri Ignatenko

**Affiliations:** 1 Scientific Research Institute, Regional Clinical Hospital #1 NA Prof. S.V. Ochapovsky, 1st May Street 167, 350086 Krasnodar, Russia; tarlan.bakhishev@yandex.ru (T.B.); viromal@mail.ru (R.V.); aslan.zakeryaev@gmail.com (A.Z.); dr.sultan@inbox.ru (S.B.); eldarurakov2013@yandex.ru (E.U.); han.gerey@mail.ru (G.K.); dr.sakhno@mail.ru (L.S.); dmr.ignatenko@yandex.ru (D.I.); 2 Kuban State Medical University, Sedina Street 4, 350063 Krasnodar, Russia; pchegatlukmarina@yandex.ru

**Keywords:** limb amputation, in-hospital mortality, systemic inflammatory response index, neutrophil-to-lymphocyte ratio, hemoglobin, emergency surgery, preoperative risk model, vascular surgery, risk stratification, predictive modeling

## Abstract

**Background/Objectives**: Major limb amputation is associated with high short-term mortality, yet practical preoperative risk models remain limited. This study aimed to identify easily obtainable preoperative predictors of in-hospital mortality after limb amputation and to develop a compact predictive model. **Methods**: We retrospectively analyzed 184 adult patients undergoing major or minor limb amputation between January 2021 and July 2025 at a tertiary referral hospital. Preoperative variables included demographics, comorbidities, urgency of surgery, hemoglobin, and complete blood count-derived inflammatory indices: neutrophil-to-lymphocyte ratio (NLR), systemic inflammatory response index (SIRI), and monocyte-to-lymphocyte ratio (MLR). The primary outcome was in-hospital mortality. Multivariable logistic regression was used to construct a compact preoperative model. Model performance was assessed by area under the receiver operating characteristic curve (AUC) and calibration plots. **Results**: In-hospital mortality occurred in 36 patients (19.6%). Independent predictors in the multivariable model were emergency surgery (OR 4.39, 95% CI 1.83–10.55), age (per 1 SD; OR 1.73, 95% CI 1.16–2.59), SIRI (per 1 SD; OR 1.77, 95% CI 1.19–2.65), and hemoglobin (per 1 SD decrease; OR 0.63, 95% CI 0.43–0.91). The model demonstrated good discrimination (AUC = 0.80) and acceptable calibration. Although not included in the model, intensive care unit and total hospital length of stay were higher among non-survivors. **Conclusions**: A compact preoperative model incorporating age, urgency of surgery, hemoglobin, and SIRI provides reliable risk stratification for in-hospital mortality after limb amputation. These variables are readily available before surgery, making the model practical for bedside clinical use. Prospective multicenter validation is warranted.

## 1. Introduction

Lower-limb amputation (LLA) remains a frequent and devastating endpoint of peripheral artery disease (PAD) and diabetes despite advances in prevention, revascularization, and multidisciplinary limb-salvage pathways. Population-based cohorts consistently show substantial early and late mortality after both major and minor amputations: contemporary data from the UK National Vascular Registry report ~17% one-year mortality and ~50% five-year mortality after minor LLA, while Scandinavian and Danish nationwide series confirm persistently high case fatality after major LLA [1,2,3]. These outcomes occur against a background of rising amputation volumes in several health systems and marked international variation in practice, underscoring the need for robust prognostication and optimized perioperative care [4,5,6]. At the global level, the burden of amputation is rising. A Global Burden of Disease analysis estimated that in 2019 there were approximately 13.2 million people living with limb loss, with years lived with disability steadily increasing [7]. More recent estimates suggest that over 445 million people worldwide are affected by traumatic amputations, with a disproportionate impact on low- and middle-income countries (LMICs), where age-standardized incidence rates can exceed 60 per 100,000 population, largely due to delayed presentation, limited access to revascularization, and high infection rates [8]. By contrast, in high-income countries such as the United States, an estimated 1.6 million people were living with limb loss in 2005, with projections to reach 3.6 million by 2050, the majority involving lower extremity amputations [9].

Accurate, preoperative risk stratification is particularly valuable for counseling, shared decision-making, triage (ICU vs. ward), and tailoring perioperative monitoring. However, most existing risk tools for patients who undergo amputation are either limited to long-term outcomes, centered on major rather than mixed (major/minor) procedures, or incorporate intra/postoperative variables that are unavailable at the time of consent. Widely used scores such as POSSUM and NSQIP have demonstrated utility in general and vascular surgical populations, but their predictive accuracy in amputation cohorts is limited, and their complexity reduces feasibility in emergency settings [10,11]. Work from the UK National Vascular Registry demonstrated important clinical predictors for 30-day mortality after major LLA, but the modeling framework did not focus on CBC-derived inflammatory markers and largely targeted postoperative prediction horizons [12]. In parallel, studies in vascular surgery link routine hematological indices to outcomes after lower-extremity interventions, but translation into a compact, pre-op, amputation-specific mortality model remains incomplete [13,14].

Inflammation is a key pathobiological axis in PAD and surgical stress. Among inexpensive, universally available markers, the neutrophil-to-lymphocyte ratio (NLR) summarizes neutrophilia and relative lymphopenia—hallmarks of acute systemic inflammation and immune dysregulation. Large reviews and population studies link higher NLR to adverse outcomes across cardiovascular and surgical settings; in diabetic foot populations, elevated preoperative or baseline NLR associates with amputation risk and mortality [15,16,17,18].

Composite indices that capture multiple leukocyte lineages may add prognostic signal. The systemic inflammation response index (SIRI), calculated as (neutrophils × monocytes)/lymphocytes, was originally proposed in oncology and later validated as a prognostic marker in cardiac surgery and coronary disease [19,20,21]. Emerging data in diabetic foot care further suggest relationships between leukocyte-based composite scores and the need for amputation [22].

Given the clinical importance of early risk appraisal and the practicality of CBC-derived indices, we hypothesized that a compact panel of preoperative variables—combining clinical severity with systemic inflammatory indices—would reliably predict in-hospital mortality after limb amputation. To test this, we retrospectively analyzed consecutive patients undergoing major or minor LLA at a tertiary center, evaluated candidate pre-op predictors including NLR and SIRI, and developed/validated multivariable models focused strictly on information available before incision.

## 2. Materials and Methods

### 2.1. Study Design and Setting

This was a retrospective observational cohort study conducted at the Scientific Research Institute of the Regional Clinical Hospital No. 1 (Krasnodar, Russia), a tertiary referral center providing vascular surgery services for a population of approximately 5 million. All consecutive patients who underwent major or minor limb amputation between 1 January 2021 and 1 July 2025 were screened for eligibility. Reporting adhered to the Strengthening the Reporting of Observational Studies in Epidemiology (STROBE) guidelines [23].

### 2.2. Patient Population

Eligible participants were adults aged ≥18 years undergoing index limb amputation during the study period. Major amputations were defined as transfemoral or transtibial procedures, while minor amputations included transmetatarsal and digital amputations. Patients were excluded if (i) no complete blood count (CBC) was available within 3 h prior to surgery or (ii) hospital outcome data were missing.

#### Inclusion and Exclusion Criteria

Eligible patients were those who underwent primary or secondary LLA due to terminal atherosclerotic ischemia of the lower limb, defined as non-salvageable critical limb ischemia after exhaustion of revascularization options. Both major amputations (at or above the ankle, including below-knee and above-knee) and minor amputations (distal to the ankle, e.g., transmetatarsal, toe) were included.

The following exclusion criteria were applied:

- Amputation due to infection, sepsis, or osteomyelitis;
- Amputation due to trauma or bleeding;
- Amputation performed for malignancy or other non-atherosclerotic etiologies;
- Lack of a complete blood count (CBC) within 3 h prior to surgery.

**Figure 1:**
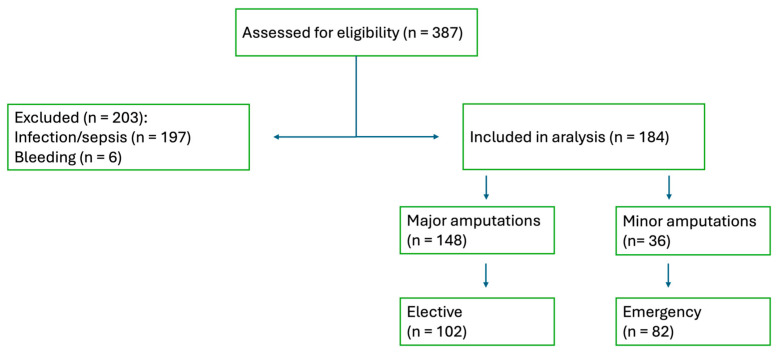
Patient flow diagram.

Demographic and clinical data were extracted from the hospital’s electronic medical record system. A total of 387 patients undergoing limb amputation were assessed for eligibility (Figure 1). After exclusion of 197 procedures due to infection or sepsis, 6 due to bleeding, and no other non-ischemic causes, 184 patients with terminal atherosclerotic ischemia were included in the final analysis. Among them, 148 underwent major amputation and 36 underwent minor amputation. Of the included cohort, 102 procedures were planned and 82 were performed as emergency surgery.

### 2.3. Variables and Definitions

Preoperative variables of interest were selected a priori:

- Demographics: Age, sex.
- Comorbidities: Smoking history (≥10 pack-years), type 2 diabetes mellitus, prior myocardial infarction, prior stroke.
- Surgical parameters: Urgency (elective vs. emergency, defined as surgery required within 24 h due to life-threatening infection, ischemia, or hemorrhage).
- Laboratory values: Hemoglobin (g/L) and CBC-derived indices.

CBC samples were obtained from peripheral venous blood collected into EDTA tubes and processed in the hospital’s central laboratory. Analyses were performed using an automated hematology analyzer (Sysmex XN-1000, Kobe, Japan). Inflammatory indices were calculated as follows:

- Neutrophil-to-lymphocyte ratio (NLR) = neutrophil count/lymphocyte count.
- Systemic inflammation response index (SIRI) = (neutrophils × monocytes)/lymphocytes.
- Monocyte-to-lymphocyte ratio (MLR) = monocytes/lymphocytes.
- Renal function parameters (serum creatinine, eGFR), nutritional indices (albumin, BMI), and frailty assessments were not systematically available in the dataset and were therefore not analyzed. The absence of these variables is acknowledged as a limitation.

Outcomes. The primary outcome was in-hospital mortality, defined as all-cause death prior to hospital discharge following the index amputation. Secondary outcomes, recorded for descriptive purposes only, included intensive care unit length of stay (ICU LOS) and total hospital length of stay (LOS).

### 2.4. Statistical Analysis

Continuous variables were expressed as median and interquartile range (IQR) and compared using the Mann–Whitney U test. Categorical variables were summarized as counts and percentages and compared using Fisher’s exact test.

Univariate logistic regression was performed to identify associations between candidate predictors and in-hospital mortality. A compact multivariable logistic regression model was constructed using only preoperative variables with clinical plausibility. Continuous predictors were standardized to z-scores to allow comparability of odds ratios per 1 SD change.

Model discrimination was evaluated by the area under the receiver operating characteristic curve (AUC) with 95% confidence intervals calculated via 1000 bootstrap resamples. Calibration was assessed using decile-based calibration plots and the Hosmer–Lemeshow test. Overall accuracy was summarized with the Brier score.

All analyses were conducted in Python 3.11 (Python Software Foundation, Wilmington, DE, USA) using the statsmodels (v0.14) and scikit-learn (v1.4) packages. Statistical significance was defined as *p* < 0.05 (two-sided).

### 2.5. Ethical Considerations

The study complied with the principles of the Declaration of Helsinki and received approval from the Institutional Review Board of the Regional Clinical Hospital No. 1. As this was a retrospective analysis of anonymized registry and laboratory data, the requirement for individual informed consent was waived.

## 3. Results

A total of 184 patients were included in the analysis. The median age of the cohort was 68.0 years [IQR 60.0–74.0], and 61.4% were male. In-hospital mortality occurred in 36 patients (19.6%).

When stratified by survival status (Table 1), non-survivors were significantly older than survivors (median 71.0 [63.8–82.0] vs. 67.0 [60.0–72.3] years, *p* = 0.013). Median hemoglobin concentration was lower in non-survivors (12.4 [10.8–14.2] g/dL) compared to survivors (13.2 [11.3–15.5] g/dL), although this difference did not reach statistical significance (*p* = 0.101). Inflammatory indices were markedly elevated in non-survivors: NLR 8.42 [5.93–16.29] vs. 4.67 [2.95–7.95], *p* < 0.001; SIRI 7.57 [4.33–12.03] vs. 3.23 [2.04–6.22], *p* < 0.001; and MLR 0.568 [0.353–0.715] vs. 0.417 [0.282–0.540], *p* = 0.0088.

Among categorical variables, the proportion of emergency surgeries was substantially higher in non-survivors (69.4% vs. 38.5%, *p* = 0.0013). A history of prior myocardial infarction and prior stroke was more frequent among non-survivors, although these differences were not statistically significant.

In univariate analysis, older age was associated with an increased risk of in-hospital death (OR = 1.58, 95% CI 1.12–2.26, *p* = 0.010). Higher NLR (OR = 2.23, 95% CI 1.55–3.21, *p* < 0.001), SIRI (OR = 2.14, 95% CI 1.53–2.99, *p* < 0.001), and MLR (OR = 1.77, 95% CI 1.28–2.45, *p* < 0.001) were also significant predictors. Lower hemoglobin was associated with higher mortality risk (OR = 0.69, 95% CI 0.49–0.97, *p* = 0.031). Emergency surgery showed the strongest association (OR = 3.75, 95% CI 1.68–8.37, *p* < 0.001) (Table 2).

**Figure 2:**
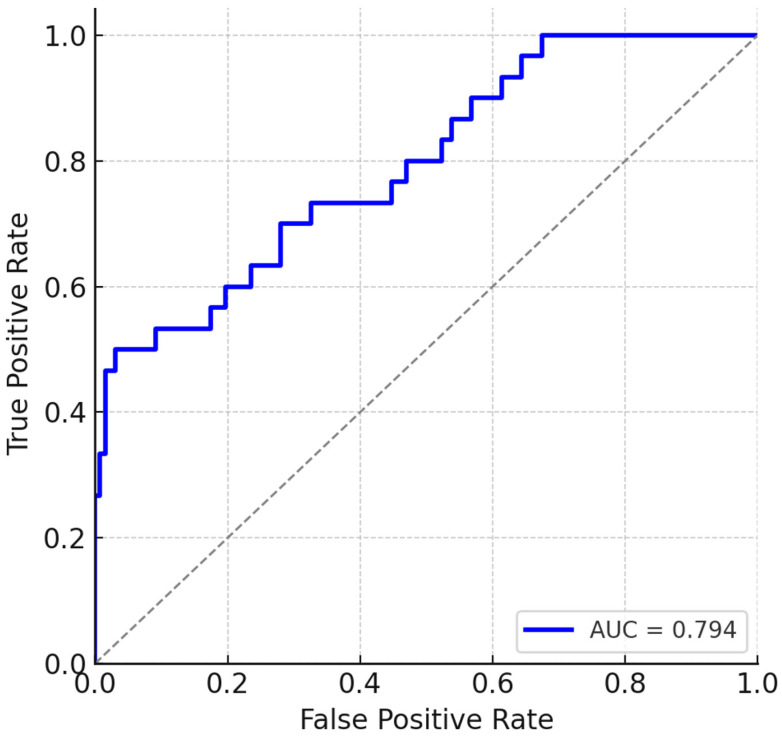
Preoperative model.

A compact preoperative model was built including all clinically relevant predictors available before surgery (Figure 2). After adjustment, the following factors remained independently associated with in-hospital mortality (Table 3):

- Emergency surgery (OR = 4.39, 95% CI 1.83–10.55, *p* = 0.001)
- Age (per 1 SD increase) (OR = 1.73, 95% CI 1.16–2.59, *p* = 0.007)
- SIRI (per 1 SD increase) (OR = 1.77, 95% CI 1.19–2.65, *p* = 0.005)
- Hemoglobin (per 1 SD decrease) (OR = 0.63, 95% CI 0.43–0.91, *p* = 0.015)

Although ICU LOS and total hospital LOS were not included in the predictive model, descriptive analysis showed that both metrics were higher among non-survivors, reflecting greater postoperative severity and complication burden in this group.

**Figure 3:**
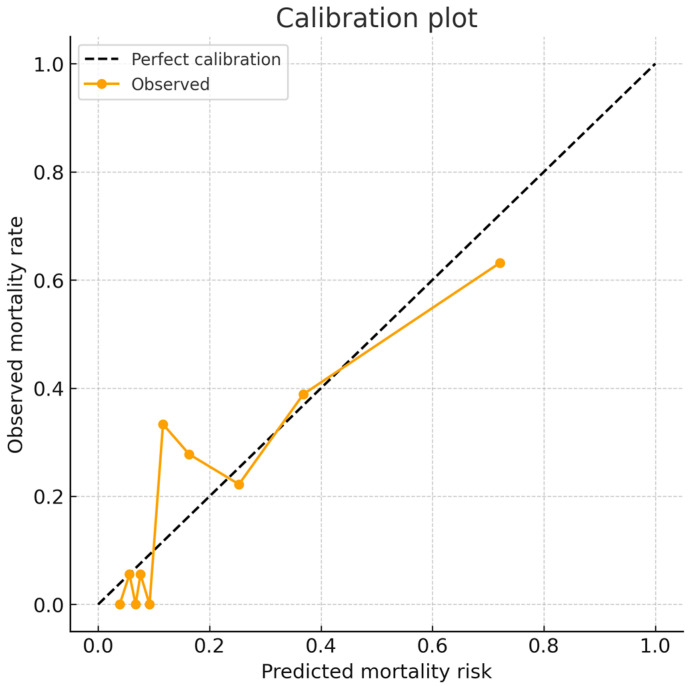
Calibration of the preoperative mortality prediction model.

The multivariable logistic regression model demonstrated satisfactory calibration (Figure 3). The Hosmer–Lemeshow goodness-of-fit test was not statistically significant (χ^2^ = 14.93, df = 8, *p* = 0.060), indicating no evidence of major miscalibration. The calibration plot (Figure 3) showed good agreement between predicted probabilities and observed event rates across deciles of risk. The overall Brier score was 0.118, consistent with acceptable probabilistic accuracy.

## 4. Discussion

In this retrospective cohort of 184 limb amputation patients, four preoperative factors—advanced age, emergency surgery, lower hemoglobin, and elevated SIRI—emerged as independent predictors of in-hospital mortality, with the compact model showing robust discrimination (AUC = 0.8). Although not part of the model, ICU and hospital LOS were substantially higher among non-survivors.

The association of emergency surgery with mortality echoes findings by Rice et al. who reported nearly double the odds of early death after urgent compared to elective amputations (OR 1.86, *p* < 0.001) [24].

Age as a risk factor aligns with broader vascular surgery data indicating progressively higher perioperative mortality with advancing age [12,25].

The strong predictive value of SIRI, a composite inflammatory index, is supported by evidence in cardiovascular surgery, where elevated SIRI was tied to poor post-operative outcomes [21], and in peripheral artery disease patients, where elevated SIRI and related indices predicted mortality [26].

While we did not derive strict cut-off values for NLR or SIRI in our dataset, several previous studies suggest potential thresholds that may be clinically relevant. For example, Afshar et al. [27] identified an NLR ≥ 6.08 as predictive of adverse outcomes, while oncology and cardiovascular cohorts have proposed SIRI thresholds in the range of 1.5–2.0. These cut-offs warrant external validation in vascular surgery populations and could inform practical risk stratification tools in the future.

Lower hemoglobin predicted higher mortality risk, consistent with literature linking anemia to impaired oxygen delivery, poor wound healing, and increased perioperative complications [28].

Interestingly, traditional cardiovascular risk factors—including smoking, prior MI, and diabetes—were not associated with in-hospital mortality in our study. This differs from some earlier reports—for example, Beeson et al. (2023), who found that age and comorbidity burden significantly predicted 5-year mortality following major lower extremity amputations [29], and Hayes et al. (2025), in which diabetes was associated with higher long-term mortality after major amputation [30]. A likely explanation is that our primary endpoint was short-term in-hospital mortality, where acute perioperative physiology (e.g., inflammation, organ support needs) may overshadow chronic baseline risks. Additionally, our high prevalence of comorbidities may limit the discriminative power of individual risk factors—a phenomenon often described as “risk saturation,” where widespread comorbidity in a cohort reduces its predictive ability. Similar saturation effects are noted in studies of surgical patients where comorbidities lose predictive power for short-term outcomes in very comorbid populations [31,32].

When comparing predictive performance, our CBC-derived model offers several advantages over established surgical risk calculators such as POSSUM and NSQIP. These established tools incorporate detailed physiological and intraoperative data, which may not be available preoperatively and can limit their applicability at the time of surgical consent. By contrast, our model relies exclusively on variables obtainable before incision—age, hemoglobin, and CBC-derived indices—making it more practical for rapid risk stratification. Nonetheless, external benchmarking against POSSUM and NSQIP in larger multicenter datasets remains a key future step.

From a pathophysiologic standpoint, neutrophil and monocyte dominance and relative lymphopenia (high SIRI) may indicate dysregulated immune response mechanisms—including endothelial dysfunction and impaired tissue regeneration—that predispose to postoperative decline, echoing concepts seen in critical care immunology and sepsis pathogenesis [33].

### 4.1. Clinical Implications

Our results have several practical applications:

1. Early identification of high-risk patients—The compact preoperative model uses only variables available before surgery and could be implemented in routine workflows to trigger enhanced monitoring or early multidisciplinary consultation.
2. Resource allocation—High-risk patients may benefit from planned ICU admission, proactive infection control measures, and more aggressive hemodynamic support.
3. Targeted research—Interventional trials focusing on modulation of perioperative inflammation, correction of anemia, and optimization of urgent surgical decision-making are warranted.

### 4.2. Strengths and Limitations

Key strengths of our study include the focus on preoperative predictors, the use of standardized laboratory and clinical data, and the application of multivariable modeling with internal performance assessment via ROC analysis. However, limitations must be acknowledged:

- The retrospective single-center design limits generalizability.
- We only assessed in-hospital mortality; longer-term outcomes were not available.
- Timing of laboratory sampling relative to surgery may have varied, potentially influencing inflammatory marker values.
- Sample size, while adequate for the primary model, may not detect small effects of less common comorbidities

### 4.3. Future Directions

Future research should focus on multicenter prospective validation of the compact preoperative model, exploration of dynamic changes in inflammatory markers during the perioperative period, and integration with other biomarkers such as high-sensitivity CRP, IL-6, and cardiac injury markers. Ultimately, these efforts could lead to the development of a validated, user-friendly risk calculator for clinical use in limb amputation populations.

Decision curve analysis (DCA) has recently been advocated as a method to evaluate the clinical utility of prognostic models by quantifying net benefit across different threshold probabilities. While DCA provides valuable insights into decision-making implications, the primary scope of our study was to identify preoperative predictors of in-hospital mortality and assess model discrimination and calibration. Therefore, we focused on ROC/AUC, Hosmer–Lemeshow test, and calibration analysis as the most widely accepted performance metrics in surgical prognostication. Future prospective studies with larger multicenter cohorts may incorporate DCA to explore the net clinical benefit of incorporating systemic inflammatory indices into perioperative decision-making tools.

## 5. Conclusions

In patients undergoing major or minor limb amputation, advanced age, emergency surgery status, lower hemoglobin concentration, and elevated systemic inflammation response index (SIRI) were identified as independent preoperative predictors of in-hospital mortality. A compact preoperative model incorporating these variables demonstrated good discriminative performance (AUC = 0.8) and can be calculated entirely from clinical and laboratory data available before surgery.

While prolonged ICU and hospital length of stay were not included in the predictive model, their markedly higher values among non-survivors highlight the role of postoperative complications in adverse outcomes. These findings emphasize the importance of early identification of high-risk patients, timely perioperative optimization, and targeted postoperative surveillance.

Prospective multicenter validation is needed to confirm these results and to assess whether integration of additional inflammatory and cardiac biomarkers can further improve risk prediction. The ultimate goal is to develop a validated, user-friendly clinical tool to guide decision-making and improve outcomes in this high-risk surgical population.

## Figures and Tables

**Table 1:** Baseline demographic and comorbidity characteristics of patients undergoing limb amputation, stratified by survival status.

Variable	Survivors (*n* = 148)	Non-Survivors (*n* = 36)	*p*-Value
Sex (male/female)	88/60	20/16	n.s.
Age, years	67.0 [60.0–72.2]	71.0 [63.8–82.0]	0.013
Hemoglobin, g/dL	13.2 [11.3–15.5]	12.4 [10.8–14.2]	0.101
Smoking ≥ 10 pack-years (yes/no)	72/76	19/17	0.707
Prior myocardial infarction (yes/no)	34/114	10/26	0.664
Prior stroke (yes/no)	27/121	11/25	0.112
Type 2 diabetes (yes/no)	99/49	27/9	0.310

**Table 2:** Univariable logistic regression for predictors of in-hospital mortality.

Variable	OR (95% CI)	*p*-Value
Age (per 1 SD)	1.58 (1.12–2.26)	0.010
Male sex	1.15 (0.55–2.42)	0.71
Smoking history	1.28 (0.61–2.69)	0.51
Prior MI	1.34 (0.58–3.07)	0.49
Prior stroke	1.41 (0.60–3.28)	0.43
Diabetes mellitus	1.22 (0.58–2.56)	0.61
Hemoglobin (per 1 SD)	0.69 (0.49–0.97)	0.031
NLR (per 1 SD)	2.23 (1.55–3.21)	<0.001
SIRI (per 1 SD)	2.14 (1.53–2.99)	<0.001
MLR (per 1 SD)	1.77 (1.28–2.45)	<0.001
Emergency surgery	3.75 (1.68–8.37)	<0.001

**Table 3:** Multivariable compact preoperative model for in-hospital mortality.

Variable	OR (95% CI)	*p*-Value
Age (per 1 SD)	1.73 (1.16–2.59)	0.007
Hemoglobin (per 1 SD)	0.63 (0.43–0.91)	0.015
SIRI (per 1 SD)	1.77 (1.19–2.65)	0.005
Emergency surgery	4.39 (1.83–10.55)	0.001

## Data Availability

The data presented in this study are available on reasonable request from the corresponding author.

## Abbreviations

CBC	Complete Blood Count
NLR	Neutrophil-to-Lymphocyte Ratio
SIRI	Systemic Inflammatory Response Index
MLR	Monocyte-to-Lymphocyte Ratio
ICU LOS	Intensive Care Unit Length of Stay
IQR	Interquartile Range
OR	Odds Ratio
AUC	Area Under the Curve
